# *Ceratopetalum* (Cunoniaceae) fruits of Australasian affinity from the early Eocene Laguna del Hunco flora, Patagonia, Argentina

**DOI:** 10.1093/aob/mcw283

**Published:** 2017-01-20

**Authors:** María A Gandolfo, Elizabeth J Hermsen

**Affiliations:** L. H. Bailey Hortorium, Plant Biology Section, School of Integrative Plant Science, 412 Mann Library Building, Cornell University, Ithaca, NY 14853, USA; Department of Environmental and Plant Biology, Porter Hall 315, Ohio University, Athens, OH 45701, USA

**Keywords:** Argentina, calibration, *Ceratopetalum*, Cunoniaceae, Eocene, fossil fruit, Laguna del Hunco, Patagonia, Schizomerieae

## Abstract

**Background and Aims:**

Radially symmetrical, five-winged fossil fruits from the highly diverse early Eocene Laguna del Hunco flora of Chubut Province, Patagonia, Argentina, are named, described and illustrated. The main goals are to assess the affinities of the fossils and to place them in an evolutionary, palaeoecological and biogeographic context.

**Methods:**

Specimens of fossil fruits were collected from the Tufolitas Laguna del Hunco. They were prepared, photographed and compared with similar extant and fossil fruits using published literature. Their structure was also evaluated by comparing them with that of modern *Ceratopetalum* (Cunoniaceae) fruits through examination of herbarium specimens.

**Key Results:**

The Laguna del Hunco fossil fruits share the diagnostic features that characterize modern and fossil *Ceratopetalum* (symmetry, number of fruit wings, presence of a conspicuous floral nectary and overall venation pattern). The pattern of the minor wing (sepal) veins observed in the Patagonian fossil fruits is different from that of modern and previously described fossil *Ceratopetalum* fruits; therefore, a new fossil species is recognized. An apomorphy (absence of petals) suggests that the fossils belong within crown-group *Ceratopetalum*.

**Conclusions:**

The Patagonian fossil fruits are the oldest known record for *Ceratopetalum*. Because the affinities, provenance and age of the fossils are so well established, this new *Ceratopetalum* fossil species is an excellent candidate for use as a calibration point in divergence dating studies of the family Cunoniaceae. It represents the only record of *Ceratopetalum* outside Australasia, and further corroborates the biogeographic connection between the Laguna del Hunco flora and ancient and modern floras of the Australasian region.

## INTRODUCTION

**Fig. 1.**
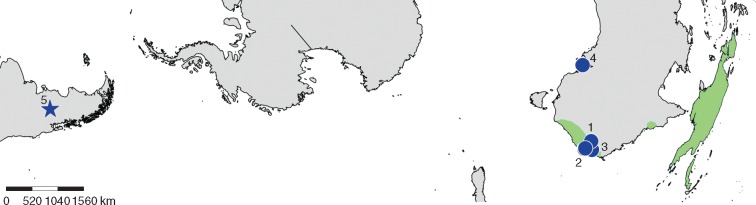
Distribution of extant *Ceratopetalum* (green) and *Ceratopetalum* fossils (blue circles, Laguna del Hunco flora marked with a blue star). Numbers adjacent to the circles on the map match the numbers in Table 1. The range of extant *Ceratopetalum* is after Hoogland (1960, fig. 1), Fortune Hopkins and Hoogland (2002, fig. 15) and Barnes *et al.* (2001, fig. 1); Australian fossil localities are from Barnes *et al.* (2001, fig. 2). The base map was produced using SimpleMappr (www.simplemappr.net).

The genus *Ceratopetalum* includes eight species found in eastern Australia, New Guinea, New Britain and several other small islands in the same region (Fig. 1; Hoogland, 1960; Fortune Hopkins and Hoogland, 2002; Rozefelds and Barnes, 2002). It is a member of the Cunoniaceae, a primarily southern hemispheric family that comprises about 27 genera, some of which are placed into six formally recognized tribes, with seven additional genera currently not included in a tribe (Bradford and Barnes, 2001; Bradford *et al.*, 2004; Sweeney *et al.*, 2004). Plants of this family are woody, with opposite or whorled leaves; they typically have bisexual flowers, often with 4–5 sepals and petals, although some members are apetalous (Fortune Hopkins and Hoogland, 2002; Bradford *et al.*, 2004). Fruits are highly variable and encompass dry dehiscent (follicles, dehiscent capsules) and indehiscent (indehiscent capsules, samaroid fruits) types, and fleshy types (drupes and berries) (Dickison, 1984; Bradford and Barnes, 2001). Morphological evolution of reproductive structures within the family, and especially of fruit types, is complex, with inflorescence, flower and fruit structure being prone to homoplasy and providing few completely unambiguous synapomorphies for the genera and tribes (Bradford and Barnes, 2001).

*Ceratopetalum* belongs within the monophyletic tribe Schizomerieae, which also includes the genera *Anodopetalum, Platylophus* and *Schizomeria* (Bradford and Barnes, 2001; Bradford *et al.*, 2004). *Anodopetalum* and *Platylophus* are monotypic (Bradford *et al.*, 2004); *Anodopetalum* is endemic to Tasmania (Barker and Brown, 1994), while *Platylophus* is restricted to south-western Cape South Africa (Dyer, 1975; Bond and Goldblatt, 1984). *Schizomeria* (approx. 10 species), like *Ceratopetalum*, is found in eastern Australia, New Guinea and other islands in the same region (Fig. 1; Hoogland, 1960; Rozefelds and Barnes, 2002; Fortune Hopkins and Hoogland, 2002). Bradford and Barnes (2001) found that the monophyly of Schizomerieae is supported by the following morphological synapomorphies: petals with incised margins; an annular floral nectary; and a heterogeneous pollen tectum. Within Schizomerieae, each genus can be distinguished in part on the basis of its fruit type. Fruits of *Anodopetalum* are interpreted as berries (Dickison, 1984) or septicidal capsules (Barnes and Rozefelds, 2000); *Schizomeria* produces drupes (Fortune Hopkins and Hoogland, 2002); and *Platylophus* produces inflated indehiscent capsules (Bradford and Barnes, 2001; Bradford *et al.*, 2004). *Ceratopetalum* has a highly distinctive indehiscent fruit with 4–6 woody wings derived from the enlarged sepals (Hoogland, 1960; Bradford and Barnes, 2001; Rozefelds and Barnes, 2002; Bradford *et al.*, 2004). Several studies have identified this fruit type and/or the semi-inferior ovary position of *Ceratopetalum* as unique apomorphies defining the genus amongst the genera of Schizomerieae (Hufford and Dickison, 1992; Bradford and Barnes, 2001; Rozefelds and Barnes, 2002); other genera of Schizomerieae have superior ovaries (Rozefelds and Barnes, 2002).

Cunoniaceae have a fossil record extending into the Cretaceous. Most cunoniaceous fossils are known from sediments of the Southern Hemisphere, although two taxa with ambiguous affinities for the family were reported from Cretaceous deposits of the Northern Hemisphere. The older of these, *Tropidogyne pikei*, was described by Chambers *et al.* (2010) based on a single flower preserved in amber from the earliest Cenomanian (Late Cretaceous) of Burma (Myanmar) and compared with *Ceratopetalum* (for revised age, see Shi *et al.* 2012). Given the age of this fossil, it seems unlikely that it represents a crown-group member of Cunoniaceae and, indeed, Chambers *et al.* (2010) suggested that the two genera might not be closely related. The younger Cretaceous taxon, *Platydiscus peltatus*, was described by Schönenberger *et al.* (2001) based on charcoalified flowers from the Santonian to Campanian (Late Cretaceous) of Sweden. Schönenberger *et al.* (2001) placed the species within Cunoniaceae but also noted a resemblance between the floral structure of *Platydiscus* and that of the unrelated rosid families Anisophyllaceae (Cucurbitales) and Cunoniaceae (Oxalidales) [see also Matthews *et al.* (2001) and Matthews and Endress (2002) on *Platydiscus*; see The Angiosperm Phylogeny Group IV (2016), for the current classification].

Confirmed fossil records of the Cunoniaceae are known only from Australia, Antarctica and South America. According to a review by Barnes *et al.* (2001), 11 modern genera of Cunoniaceae and one fossil genus (*Weinmanniaphyllum*) are reported from the fossil record of Australia beginning in the late Paleocene, where they are represented by remains of leaves, cuticles, wood and reproductive structures. Cunoniaceous pollen, although it cannot be attributed with precision to modern genera, is also known (Barnes *et al.*, 2001). Outside of Australia, the fossil record of the family is generally recognized on the basis of wood and pollen. Several species based on wood specimens have been described from the Cenomanian to early Campanian of King George Island (Isla 25 de Mayo) and Livingston Island, South Shetland Islands, Antarctica (Torres, 1985; Poole *et al.*, 2000, 2001). Additional reports come from the Paleogene to Miocene of Chilean and Argentinean Patagonia (Petriella, 1972; Brea et al., 2004, 2012, 2015; Terada *et al.*, 2006). Fossil pollen grains with affinities for Cunoniaceae are known from the Late Cretaceous to late Paleocene of the Antarctic Peninsula (Cranwell, 1959; Askin, 1992) and the late Paleocene to mid-Miocene of Patagonia (Petriella and Archangelsky, 1975; Romero and Castro, 1986; Barreda *et al.*, 2007). In the Quaternary, pollen is known from deposits in Argentina and Chile (e.g. Heusser, 1974; Arbazúa *et al.*, 2004), as well as other countries in South America, such as Colombia (e.g. Schreve-Brinkman, 1978; Hooghiemstra, 1989). The fossil record of *Ceratopetalum* was heretofore restricted to Australia, where it is based solely on fossil fruits of Eocene to Miocene age (Table 1; Fig. 1).

**Table 1:** Fossil record of *Ceratopetalum*

Species	Age	Locality/formation	State/country	Reference
*C. priscum* Holmes & Holmes (1)	Middle Miocene	Quarry H and A/Chalk Mountain	New South Wales, Australia	Holmes and Holmes (1992); Barnes and Hill, (1999)
*C. westermannii* R.W. Barnes & R. S. Hill (2)	Early Miocene	Elands	New South Wales/Australia	Barnes and Hill (1999)
*C. wilkinsonii* (Ett.) Holmes & Holmes (3)	Late Eocene–early Oligocene	Old Rose Valley Lead	New South Wales/Australia	Holmes and Holmes (1992); Barnes and Hill, (1999)
*C. maslinensis* R.W.Barnes & R.S.Hill (4)	Middle Eocene	Maslin Bay/North Maslin Sand	South Australia/Australia	Barnes and Hill (1999)
*C. edgardoromeroi* Gandolfo & Hermsen (5)	Early Eocene	LH4, LH6 and LH25/Tufolitas Laguna del Hunco	Chubut, Argentina	This study

Additional reports considered invalid by Barnes, Hill and Bradford (2001) have been omitted.

Numbers following species names correspond to the locality numbers in Fig. 1.

In this contribution, we report radially symmetrical, five-winged fossil fruits with unique characters supporting their inclusion within the genus *Ceratopetalum*. These fossils come from the early Eocene Tufolitas Laguna del Hunco flora, Chubut Province, Patagonia, Argentina (Fig. 1). They are significant because they represent the first record of Cunoniaceae based on reproductive macrofossils from South America, the oldest unambiguous record for cunoniaceous reproductive macrofossils worldwide and the first known occurrence of the genus *Ceratopetalum* (extant or fossil) outside of Australasia.

## MATERIALS AND METHODS

The Tufolitas Laguna del Hunco belongs to the Middle Río Chubut Volcanic–Pyroclastic Complex (Aragón and Mazzoni, 1997; Aragón *et al.*, 2004). Sediments of the Tufolitas Laguna del Hunco outcrop in north-western Chubut Province, Patagonia, Argentina (Fig. 1); they represent tuffaceous lacustrine deposits that have yielded one of the most species-rich Eocene macrofloras yet discovered (Wilf *et al.*, 2003, 2005). The fossil fruits were recovered from three (LH4, LH6 and LH25) of a total of 27 quarried localities (see Wilf *et al.*, 2003 for coordinates). The age for all the localities is Ypresian or early Eocene [approx. 52 million years ago (Ma)] as calculated by radiometric and palaeomagnetic methods (see Wilf *et al.*, 2003, 2005; and Wilf, 2012, for additional discussion of the age). The fossils were trimmed in the field, and additional preparation was performed by staff of the Museo Paleontológico Egidio Feruglio (MEF; Trelew, Chubut Province, Argentina).

Specimens described herein are housed in the palaeobotanical collection of the MEF (specimen number prefix MPEF-Pb).

Extant *Ceratopetalum* material used for comparison to the fossil specimens comes from herbarium sheets that are held at the L. H. Bailey Hortorium (BH), Plant Biology Section, School of Integrative Plant Science, Cornell University, Ithaca, NY, USA and the National Herbarium of Victoria, Royal Botanic Gardens Melbourne (MEL), Victoria, Australia. Fossils were photographed using a Nikon D70 Digital SLR camera at BH and MEF. Images of extant *Ceratopetalum* were taken at MEL using an Epson Expression 10000 XL, Model J181A scanner mounted in a HerbScan framework, and at BH with a Nikon D800e camera. Plates were prepared using Adobe Photoshop CS4 Extended ver. 11.0 (1990–2008 Adobe Systems Inc.), and line drawings of the venation patterns of the sepals of the fossil and extant *Ceratopetalum* and schematic diagrams of venation patterns were produced with Adobe Illustrator Creative Cloud 2015.0.0 Release (1987–2015 Adobe Systems Inc.).

## RESULTS

*Family*. Cunoniaceae R. Br. 1814

*Tribe*. Schizomerieae J. C. Bradford & R. W. Barnes 2001

*Genus*. ***Ceratopetalum*** Sm. 1793

*Species*. ***Ceratopetalum edgardoromeroi*** Gandolfo & Hermsen*, sp. nov.*

cf. Cunoniaceae, sp. no. TY129 (LH4-169 = MPEF-Pb 5087), Wilf *et al.* (2005), appendix table 2.

**Table 2:** Floral characters of *Ceratopetalum* species

	No. of wings	Wing shape	Wing apex	Wing base	Length (mm)	Width (mm)	Petals	Nectary disc	‘Ovary’ diameter (mm)	Stamens on fruit
*gum*	4–6	Narrowly to broadly obovate	Acute	Not constricted	9·8–16·1	2·7–6·7	Present	Present	3·4–4·8	Present
*ape*	4–6	Obovate to ovate	Acute	Constricted	6·3–8·9	2·1–4·3	Absent	Present	3–4·4	Present
*cor*	4–6	Obovate	Acute	Slightly constricted	At least 7	?	Absent	Present	?	?
*hyl*	4	Narrowly obovate to lanceolate	Acute to obtuse	Slightly constricted	6·6–11·2	2·2–3	Absent	Present	3·6–4	Present
*suc*	4–5	Elliptical to obovate	Acute	Slightly constricted	8·3–12·6	2·4–4·1	Absent	Present	3·6–4·2	Present
*vir*	4–6	Obovate to lanceolate	Acute	Constricted	11·5–13·5	3·4–4·9	Absent	Present	6·1–7·3	Present
*tet*	4	Ovate to obovate	Acute	Slightly constricted or not	8·8–17	3·8–5·1	Absent	Present	6–8	Present
*iug*	4	Narrowly to broadly obovate	Acute	Not constricted	15	4·8–5	Absent	Present	?	Present
*mac*	4–5	Obovate to lanceolate	Acute	Constricted	10–13	3·2–4·5	Absent	Present	5·4–5·9	Present
*pri	5	Narrow oblong	Obtuse	Not constricted	7–10	3–4	Present	Present	3–5	Absent
*wes	5	Narrow obovate	Rounded	Not constricted	6–9	1·8–2·2	Absent	Absent?	1–1·2	Present
*mas	5–6	Narrow oblong	Acute to obtuse	Not constricted	5–5·5	∼1·5–2	Absent	Absent?	∼2·1	Absent
*wil	5	Ovate to elliptical	Obtuse	Not constricted	∼10	4–5·5	Present	Present	6·5	Absent
*edg	5	Narrow obovate	Rounded	Constricted	10	2–4	Absent	Present	4–5	Absent

*gum, C. gummiferum*; *ape, C. apetalum*; *cor, C. corymbosum*; *hyl, C. hylandii*; *suc, C. succirubrum*; *vir, C. virchowii*; *tet, C. tetrapterum*; *iug, C. iugumensis*; *mac, C. macrophyllum*; *pri, C. priscum; *wes, C. westermannii; *mas, C. maslinensis; *wil, C. wilkinsonii; *edg*, *C. edgardoromeroi.

* Denotes fossils.

Data for extant species are gathered from Hoogland (1960), Fortune Hopkins and Hoogland (2002), and Rozefelds and Barnes (2002); data for fossil species from Holmes and Holmes (1992), Barnes and Hill (1999) and this contribution.

All measurements are given in mm.

Unknown dicot sp., sp. no. TY145 (LH6-1017 = MPEF-Pb 5085), Wilf *et al.* (2005), appendix table 2.

*Specific diagnosis*. Fruit with five wings (sepals) surrounding a circular central region. Each wing obovate, apex rounded and base constricted, vascularized by three primary veins; midvein prominent and two lateral veins weaker; primary veins branching in the distal two-thirds of each wing, branches sometimes anastomosing to form a partially closed reticulum. Petals absent. Annular nectary present, prominent.

**Fig. 2.**
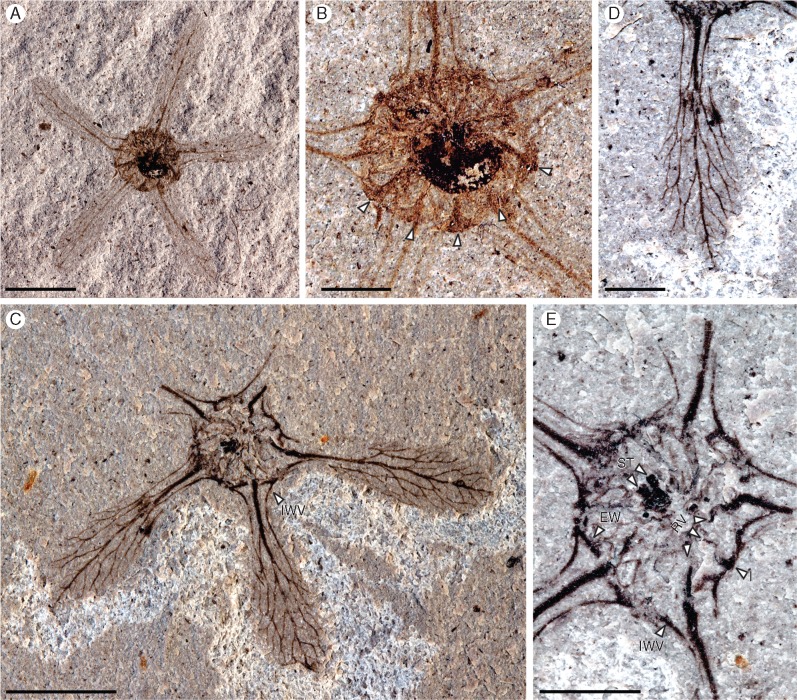
*Ceratopetalum edgardoromeroi* Gandolfo and Hermsen, *sp. nov*. (A) Overall view of the holotype showing the five wings and the circular central region with nectary and semi-inferior ovary. MPEF-Pb 5085a. Scale bar = 10 mm. (B) Detail of the central region showing the nectary and radiating veins (arrows show examples). MPEF-Pb 5085a. Scale bar = 2 mm. (C) Overall view of the specimen showing three complete or nearly complete wings and two wing bases, prominent nectary, central styles marking the position of the ovary and inter-wing veins (IWV). MPEF-Pb 5086b. Scale bar = 5 mm. (D) Detail of fruit wing showing three primary veins emerging from the central region and branching distally to form a reticulum of minor veins. MPEF-Pb 5086a. Scale bar = 5 mm. (E) Detail of the circular central region showing ends of radiating veins (RV), inter-wing veins (IWV), intersections of the inter-wing and radiating veins (I) and well-preserved encircling vein (EV) crossing beneath a wing. Styles (ST) are visible in the centre of the nectary. MPEF-Pb 5086b. Scale bar = 5 mm.

*Holotype designated here*. MPEF-Pb 5085a, b (LH6), Fig. 2A, B. Repository, Museo Paleontológico Egidio Feruglio (MEF).

*Type locality, age and stratigraphy*. Laguna del Hunco paleoflora locality LH6, Ypresian (early Eocene) Tufolitas Laguna del Hunco, Chubut Province, Argentina.

*Additional material examined*. MPEF-Pb 5084 (LH4), MPEF-Pb 5087 (LH4) and MPEF-Pb 5086a, b (LH25).

### Etymology

The species epithet ‘*edgardoromeroi*’ is erected in honor of Edgardo J. Romero, a prestigious Argentinean palaeobotanist, for his many contributions to our understanding of the angiosperm palaeofloras of Patagonia, Argentina.

### Species description

The fruits are radially symmetrical, apparently indehiscent and lacking petals. They are 2·3–2·6 cm in diameter. Each fruit has a calyx of five persistent wings (sepals) attached to a circular central region (Fig. 2A, C). Each wing is narrowly obovate with a rounded apex, a slightly constricted base and an entire margin (Fig. 2A, C); wing dimensions are approx. 1 cm long and 0·2–0·4 cm wide. Each wing is vascularized by three primary veins, a prominent midvein and two less prominent lateral veins (Fig. 2A, C, D). The midvein radiates from within the central region of the fruit and proceeds in a slightly sinuous pattern to the tip of the wing, giving off lateral branches (Fig. 2A, C, D). Each lateral vein is continuous basally, with the nearest lateral vein in the adjacent wing, forming an inter-wing vein that follows the edge of the central region (Fig. 2C, E). Each lateral vein also branches distally, becoming indistinct in its course as it does so (Fig. 2C, D). The branches of the primary veins anastomose occasionally, yielding a reticulum in the distal two-thirds of the wing (Fig. 2C, D).

The circular central region of the fruit is 0·5–0·6 cm in diameter and has two distinct zones, a thick outer ring and an inner circular zone (Fig. 2A–C, E). The thick outer ring is interpreted as representing an annular nectary and is 0·1–0·2 cm wide; the inner circular zone is interpreted as representing the upper part of the ovary and is 0·4–0·5 cm in diameter (Fig. 2A–C, E). Thick veins radiating from the central region of the fruit are visible on and at the edge of the nectary (Fig. 2A–C, E). Some of these radiating veins are the veins that continue into the wings to become the wing midveins (Fig. 2A–C, E); alternating with these are radiating veins that fuse with each inter-wing vein at its midpoint (Fig. 2B, E). Since one radiating vein that proceeds into each wing alternates with one radiating vein that fuses with an inter-wing vein (Fig. 2B, E), the fruit is interpreted as having had ten radiating veins.

A prominent vein crosses the base of a wing near the outer edge of the nectary on one specimen; this vein intersects the wing midvein at a right angle and merges with the inter-wing veins on either side (Fig. 2C, E). Given the symmetry of the fruit and the remnants of what appear to be additional, similar veins near the outer edge of the nectary, the fruit may have had a thick vein proceeding across the base of each wing and fusing with the interwing veins on either side, thus forming a continuous series of veins encircling the periphery of the nectary.

The nectary region shows remnants of tissue, perhaps including vascular tissue forming thin veins, although no particular venation pattern was observed beyond that described above (Fig. 2A–C, E). One specimen shows at least two styles, although four structures may be present (Fig. 2C, E), indicating that there were either two unlobed styles, two bilobed styles or four styles; thus, carpel number is inferred to be two or four. Based on the prominence of the annular nectary relative to the ovary (Fig. 2A–C, E), the ovary is interpreted as at least semi-inferior.

## DISCUSSION

### Assignment to *Ceratopetalum* and recognition of new species

**Fig. 3.**
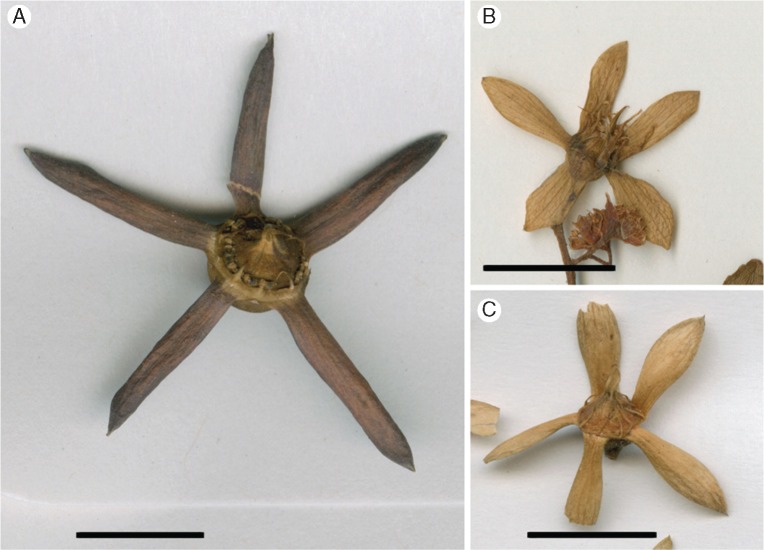
Extant *Ceratopetalum* fruits. (A) *C. macrophyllum*. MEL2277402. (B) *C. gummiferum*. MEL648233. (C) *C. apetalum*. MEL2048597. Scale bar = 1 cm.

The floral and fruit morphology of extant *Ceratopetalum* have been described in detail by, for example, Hoogland (1960), Dickison (1984), Barnes and Hill (1999), Matthews *et al.* (2001), Fortune Hopkins and Hoogland (2002) and Rozefelds and Barnes (2002). Extant *Ceratopetalum* species produce fruits with a radially symmetrical, persistent calyx of 4–6 wings (enlarged sepals) attached to a central receptacle (Fig. 3A–C). Stamens are persistent on the fruits and are found both alternating with and opposite the wings (Fig. 3A–C). The ovary is semi-inferior, indehiscent and composed of two or sometimes three carpels; a conspicuous annular nectary surrounds the upper portion of the ovary in the flowers (see, for example, fig. 5 in Rozefelds and Barnes, 2002), although its appearance is less pronounced in the fruit (Fig. 3A, C).

**Fig. 4.**
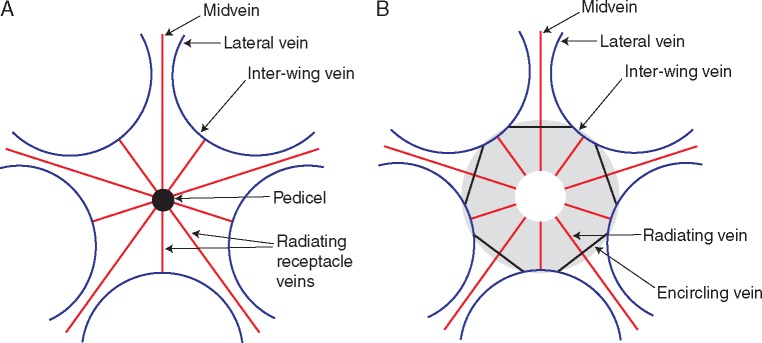
Schematic diagrams showing fruit venation patterns of *Ceratopetalum*. (A) Extant *Ceratopetalum*. (B) *C*. *edgardoromeroi*. The shaded area represents the region interpreted as the nectary in the fossil fruits. The central white circle represents the region of the ovary. While it is presumed that the radiating veins would have passed beneath the ovary to the fruit pedicel as in extant *Ceratopetalum* (Barnes and Hill, 1999), this has been left intentionally ambiguous in the drawing since it was not observed. Encircling veins are here reconstructed as crossing beneath each wing.

According to Barnes and Hill (1999), extant *Ceratopetalum* flowers and fruits share a similar venation pattern, as reconstructed here (Fig. 4A) based on descriptions by Dickison (1975), Barnes and Hill (1999) and Matthews *et al.* (2001), as well as fig. 3 of Barnes and Hill (1999): each sepal/fruit wing is fed by three primary veins that emerge from the receptacle (i.e. extra-ovarian floral tissue fused to the ovary wall), a midvein with one lateral vein on either side; additional, less prominent lateral wing veins may also be present in the wing bases of some species. The lateral primary veins from adjacent wings are continuous basally, forming inter-wing veins. Prominent veins radiating from the receptacle to form the wing midveins alternate with veins that fuse with the inter-wing veins near the edge of the receptacle. The number of veins radiating from the receptacle of the flower is thus twice the number of fruit wings, or equal to the number of stamens.

The three-veined sepals and inter-sepal veins formed by the fusion of lateral veins from adjacent sepals are characteristics shared across flowers of nearly all genera of Cunoniaceae that have been examined (Dickison, 1975; Barnes and Hill, 1999; Matthews *et al.*, 2001; Bradford *et al.*, 2004). Therefore, flower or fruit venation pattern alone is not necessarily diagnostic for the genus, but must be considered in conjunction with other features.

*Ceratopetalum edgardoromeroi* fruits are largely consistent with extant *Ceratopetalum* in their morphology and venation pattern. *Ceratopetalum edgardoromeroi* is a five-winged fruit with wings attached to a circular region interpreted here as consisting of a central ovary surrounded by a prominent annular nectary (Fig. 2A–C, E). Due to the prominence of the nectary in relation to the relatively small region interpreted as representing the gynoecium (Fig. 2A–C, E), the Patagonian fossils are interpreted as probably having had a semi-inferior ovary, although the position of the ovary cannot be definitively confirmed due to the orientation in which the fruits are preserved. Based on the presence of 2–4 structures interpreted as styles on one specimen (Fig. 2C, E), *C*. *edgardoromeroi* is interpreted to be bicarpellate or tetracarpellate; although bicarpellate flowers are a synapomorphy for a large clade of Cunoniaceae including tribe Schizomerieae, flowers with more than two carpels have originated separately several times, including within Schizomerieae (Bradford and Barnes, 2001). *Ceratopetalum edgardoromeroi* fruits do not have stamens preserved, which conflicts with the fruits of modern *Ceratopetalum* species. Lack of persistent stamens is, however, consistent with other fossil fruits assigned to *Ceratopetalum*, except for *C. westermannii* (Table 2).

The venation pattern of the fruits, preserved in two specimens (Fig. 2A–E), is similar to that described for extant species (cf. Fig. 4A and B). Wings have three primary veins, and lateral primary veins from adjacent wings fuse to form inter-wing veins (Figs 2A–C, E and4B). Veins radiate from the circular region at the centre of the fruit, continuing into the wings or ending at the inter-wing veins (Figs 2A–C and4B). In one specimen, these veins appear to widen at the periphery of the nectary, perhaps representing the attachment points of the stamens (Fig. 2A, B). Although the radiating veins are observed in or at the margins of the nectary in *C*. *edgardoromeroi*, they are assumed to be passing beneath the nectary proper, presumably through tissue beneath or surrounding the ovary (i.e. the receptacle). Large veins crossing the bases of the wings and partially or wholly encircling the periphery of the nectary in *C*. *edgardoromeroi* (Figs 2C, E and 4B) are one feature not noted as characterizing the extant genus. These veins may be associated with the nectary or, more probably, the rim of the receptacle or even the base of the calyx. The nectary also shows the remnants of thick tissue or perhaps minor veins (Fig. 2A–C, E); Matthews *et al.* (2001) noted phloem in the nectary of extant *C*. *gummiferum* flowers.

Within Cunoniaceae, the only other taxon that produces fruit with five wings formed by a persistent calyx is *Pullea* (tribe Codieae); this taxon, like *Ceratopetalum*, also has a semi-inferior ovary (Fortune Hopkins and Hoogland, 2002), which is relatively uncommon amongst genera of Cunoniaceae (Bradford and Barnes, 2001). The similarities in fruit morphology between *Ceratopetalum* and *Pullea* were interpreted as likely to be the result of convergent evolution (Bradford and Barnes, 2001). *Pullea* is native to Fiji, Australia, New Guinea and other small islands north of Australia as far north as Morotai (Hoogland, 1979; Fortune Hopkins and Hoogland, 2002). According to Fortune Hopkins and Hoogland (2002), *Pullea* can be distinguished from *Ceratopetalum* by its imbricate sepal aestivation, a feature that can apparently be inferred even from the fruiting stage; in contrast, *Ceratopetalum* flowers have valvate sepal aestivation (Rozefelds and Barnes, 2002). Additionally, *Pullea* fruits have papery wings and relatively long, persistent styles (Fortune Hopkins and Hoogland, 2002).

**Fig. 5.**
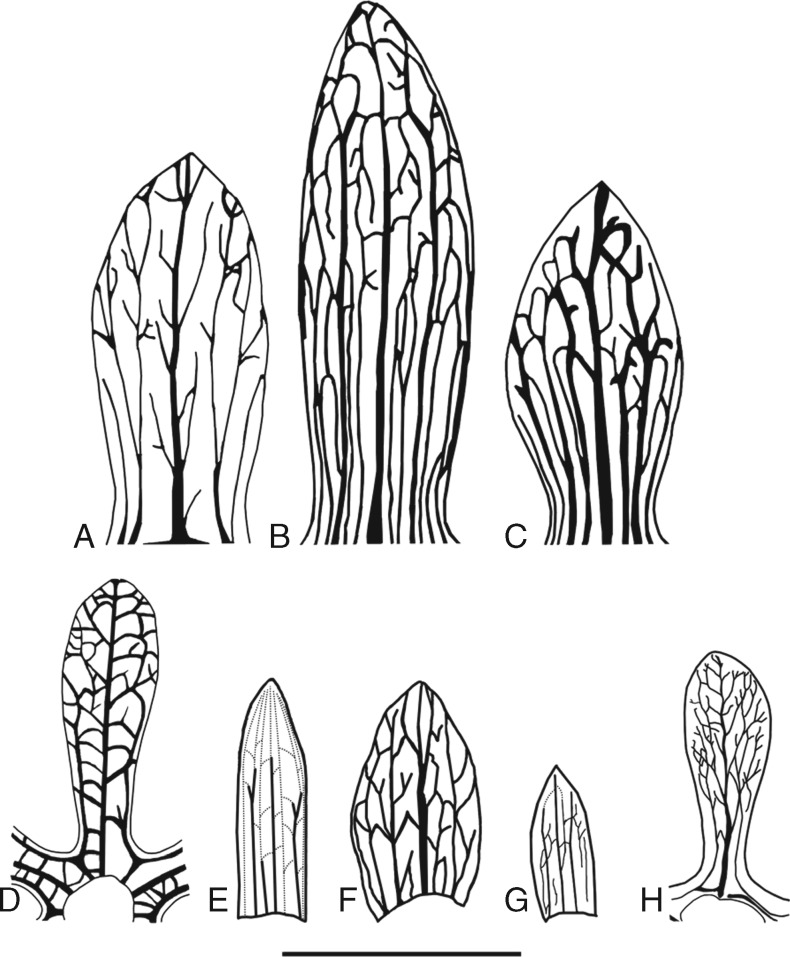
Line drawing of wing morphology and venation pattern of selected extant and all fossil species of *Ceratopetalum*. (A–C) Extant species. (A) *C. gummiferum*. (B) *C. succirubrum*. (C) *C. virchowii*. (D–H) Fossil species. (D) *C. westermannii*. (E) *C. priscum*. (F) *C. wilkinsonii*. (G) *C. maslinensis*. (H) *C. edgardoromeroi*. See Tables 1 and 2 for descriptions; (A–D) are redrawn from Barnes and Hill (1999). Scale bar = 1 cm.

Extant and fossil *Ceratopetalum* species can be distinguished by certain fruit characteristics, including a combination of wing (sepal) number, wing morphology, wing venation pattern and presence or absence of petals and stamens on mature fruits (Table 2).*Ceratopetalum edgardoromeroi* can be distinguished from other known species by a combination of wing number (five), wing shape (obovate with rounded apex and constricted base), absence of petals and stamens, and wing venation. The partially closed reticulum formed by the minor veins in particular seems to be unique for this species among fossil and extant *Ceratopetalum* (Fig. 5A–H).

### Character evolution and age of *Ceratopetalum*

*Ceratopetalum edgardoromeroi* is typical of fossil *Ceratopetalum* fruits in having five wings (sepals) and in lacking stamens (Table 2). Based on a morphological phylogenetic analysis of Schizomerieae focusing on intrageneric relationships of *Ceratopetalum*, Rozefelds and Barnes (2002) found that tetramerous flowers are derived within the genus, and that they had originated twice: once in *C*. *iugumensis* and once in a clade including *C. hylandii, C*. *succirubrum* and *C*. *tetrapterum*. Thus, all fossils appear to have the plesiomorphic condition for perianth merosity. All extant *Ceratopetalum* species (except *C*. *corymbosum*, in which this character has not been documented) retain whole stamens or remnants of stamens on their fruits (Fig. 3A–C; Rozefelds and Barnes, 2002), whereas all fossil species, except *C. westermannii* (early Miocene), lack them (Holmes and Holmes, 1992; Barnes and Hill, 1999; this study). This would suggest either that the stamens were routinely lost during preservation of *Ceratopetalum* fossils, or that persistence of the stamens on the fruits is a novel structural feature that appeared some time during the early Miocene.

Only three species of *Ceratopetalum* have a corolla: the extant *C. gummiferum* (Fig. 3B) and the Australian fossil fruits *C. priscum* and *C. wilkinsonii* (Holmes and Holmes, 1992; Barnes and Hill, 1999; Rozefelds and Barnes, 2002). If the absence of petals in the fossil fruits is interpreted as a true absence of petals as in most extant species of *Ceratopetalum*, the remaining species lack petals (Table 2). Petals with incised margins are a synapomorphy for the tribe Schizomerieae as a whole (Bradford and Barnes, 2001), whereas the absence of petals is thought to be apomorphic within *Ceratopetalum* (Rozefelds and Barnes, 2002). The lack of petals in *C. edgardoromeroi*, now the oldest documented occurrence of fossils assignable to *Ceratopetalum*, would thus indicate that the loss of petals within the genus occurred before the early Eocene. Previously, the loss of petals was considered to have dated minimally to the middle Eocene, as evidenced by lack of petals in *C*. *maslinensis* (Rozefelds and Barnes, 2002). Lack of petals in *C*. *maslinensis* was also previously considered to indicate that *Ceratopetalum* itself had originated prior to the middle Eocene (Rozefelds and Barnes, 2002). Since *C*. *edgardoromeroi* likewise lacks petals, suggesting that it nests within crown-group *Ceratopetalum*, the age of the genus must now be considered to date minimally to the early Eocene.

Criteria for choosing fossils to use in divergence dating analyses have been suggested by Gandolfo *et al.* (2008) and Parham *et al.* (2012). The age of the *C*. *edgardoromeroi* specimens is well constrained, the source locality of the fossils is well documented, the identity of the fossils is well founded on the basis of their overall structure and an apomorphy (lack of petals) convincingly places them within crown-group *Ceratopetalum*; thus, *C*. *edgardoromeroi* would make a suitable calibration point for divergence dating analyses. The LH4 locality occurs below a stratigraphic layer most recently dated as 52·22 ± 0·29 Ma (Wilf *et al.*, 2003, 2005; Wilf, 2012), making the numerical minimum age currently associated with *C*. *edgardoromeroi* 51·93 Ma. Notably, in addition to being the oldest fossil record of *Ceratopetalum, C*. *edgardoromeroi* is the oldest fossil record for tribe Schizomerieae.

### Palaeoecology and palaeobiogeography

Extant *Ceratopetalum* species have a disjunct distribution confined to Australasia (Fig. 1). In Australia, *Ceratopetalum* is found in sub-tropical to warm temperate rain forests of eastern New South Wales and south-eastern Queensland, as well as north-eastern Queensland (Baur, 1957, 1979; Hoogland, 1960; Floyd, 1990; Rozefelds and Barnes, 2002). Only one species grows outside Australia, occurring in New Britain, New Guinea and small islands nearby (Fortune Hopkins and Hoogland, 2002; Rozefelds and Barnes, 2002), where it has been documented from sub-tropical rain forests (Takeuchi, 1999*a*, *b*, 2002; Paul, 2011). *Ceratopetalum* shows a biogeographic pattern typical of the Laguna del Hunco flora, since the genus is extinct in South American today but is a component of the Paleogene to recent rain forest vegetation of Australasia (see, for example, Wilf *et al.*, 2009, 2014; Kooyman *et al.*, 2014; Merkhofer *et al.*, 2015).

The palaeocommunity in which *C. edgardoromeroi* grew was composed of pteridophytes, including *Dicksonia, Sticherus, Todea* and Pteridaceae (Carvalho *et al.*, 2010, 2013); conifers, such as *Agathis, Dacrycarpus* and *Papuacedrus* (Wilf *et al.*, 2009, 2014; Wilf, 2012); the ginkgophyte *Ginkgoites* (Villar de Seoane *et al.*, 2015); and angiosperms, including magnoliids (Atherospermataceae and Monimiaceae: Knight and Wilf 2013), eudicots (other Cunoniaceae, *Akania, Eucalyptus, Gymnostoma*, Juglandaceae and several Proteaceae: Romero and Hickey, 1976; Gandolfo *et al.*, 1988, 2011; Zamaloa *et al.*, 2006; González *et al.*, 2007; Gandolfo and Hermsen, 2012; Hermsen *et al.*, 2012; Hermsen and Gandolfo, 2016), and the monocot *Ripogonum* (Carpenter *et al.*, 2014). This palaeocommunity is similar in composition to modern rain forest communities of Australia and New Guinea where extant *Ceratopetalum* occurs. *Ceratopetalum* is dominant in some rain forest alliances in Australia (see Baur, 1957, 1979; Burges and Johnston, 1953; Floyd, 1990; Kooyman *et al.*, 2014), while it is typically an occasional component of plant communities in Papua New Guinea (Takeuchi, 1999*a*, 2002; Paul, 2011). The few specimens (four specimens in total) of C. *edgardoromeroi* found in the Laguna del Hunco flora suggest that it was not a prominent component of the Laguna del Hunco palaeocommunity, but rather relatively rare.

### Conclusions

In their paper on the evolution of angiosperm fruits, Collinson and van Bergen (2004) separated winged fruits into several categories based on wing position. Since the Patagonian fossils have multiple wings, they belong to Collinson and van Bergen’s (2004: p. 377) category ‘Multiple wings (‘helicopters/propellers’)’, which includes fossil fruits with at least three (and typically more) wings oriented in a more or less horizontal position. Within this category, nine genera have a Paleogene fossil record (Table 3): *Asterocarpinus* (Betulaceae), *Ceratopetalum* (Cunoniaceae), *Chaneya* (probably within Simaroubaceae), *Cruciptera* (Juglandaceae), *Tetrapterys* (Malpighiaceae), *Trilobium* (Anacardiaceae?), and *Calycites, Ozakia* and *Raskya* (affinity uncertain). With the exception of *Ceratopetalum*, all genera representing multiwinged helicopter-type fruits are known from Northern Hemisphere palaeofloras of Europe, eastern Asia and/or western North America (Table 3). This report of *Ceratopetalum* is the first documented occurrence of a multiwinged helicopter-type fruit genus showing an intercontinental connection between Southern Hemisphere continents, and the first Paleogene report of this fruit type from South America. The helicopter-type Laguna del Hunco fruits described in this study clearly represent a new record of fossil *Ceratopetalum*: they have five wings, an annular floral nectary, a semi-inferior ovary and a floral/fruit venation pattern consistent with Cunoniaceae and *Ceratopetalum* (Fig. 4A, B).

**Table 3:** Paleogene multiwinged, helicopter-type fruits (see Collinson and van Bergen, 2004)

Genus	Family	Continent	Age	Publication(s)
*Asterocarpinus*	Betulaceae	North America	Late Eocene–early Oligocene	Manchester and Crane (1987); Meyer and Manchester (1997)
*Calycites*	Unknown	Europe	Paleocene	Crane (1988)
		North America	Paleocene–middle Eocene	Crane (1988); Wehr (1995)
*Ceratopetalum*	Cunoniaceae	South America	Eearly Eocene	This study
		Australia	Middle Eocene–middle Miocene	Holmes and Holmes (1992); Barnes and Hill (1999)
*Chaneya*	Simaroubaceae?	North America	Middle Eocene–? late Oligocene	Wang and Manchester (2000)
		Asia	Late Eocene–middle Miocene	Wang and Manchester (2000)
*Cruciptera*	Juglandaceae	Europe	Middle Eocene	Manchester (1991, 1999); Manchester *et al.* (1994)
		North America	Middle Eocene–Oligocene	Manchester (1991, 1999, 2000); Wehr (1995); Meyer and Manchester (1997)
*Ozakia*	Unknown	North America	?Oligocene–Miocene	Manchester and Uemura (2014)
		Asia	Late Miocene	Manchester and Uemura (2014)
*Raskya*	Unknown	Europe	Late Eocene–early Oligocene	Manchester and Hably (1997); Thiebaut (1999)
*Tetrapterys*	Malpighiaceae	Europe	Early Oligocene	Hably and Manchester (2000)
*Trilobium*	Anacardiaceae?	Europe	Middle Eocene–Oligocene	Wilde and Frankenhäuser (2010)

The lack of petals on *C*. *edgardoromeroi* suggests that they belong within the crown-group for the genus. The fruits are assigned to a new species, *C*. *edgardoromeroi*, primarily on the basis of the venation pattern of their wings, which differs from that described for other fossil and modern *Ceratopetalum* fruits (Fig. 5A–H). Due to their well-documented geographic and stratigraphic provenance, as well as the features establishing their position within the genus *Ceratopetalum*, the fossils provide a robust minimum age for the genus of 51·93 Ma. *Ceratopetalum edgardoromeroi* fits comfortably into the biogeographic pattern already established for the Laguna del Hunco flora, as the genus *Ceratopetalum* has previously been described only from Australasia.
